# Underwater Robot Object Detection Algorithm Based on YOLOv11

**DOI:** 10.3390/s26113611

**Published:** 2026-06-05

**Authors:** Yongqing Shi, Wei Chen, Duo Wan, Lu Han

**Affiliations:** School of Automation, Jiangsu University of Science and Technology, Zhenjiang 212003, China; 241210302110@stu.just.edu.cn (Y.S.); 241110302103@stu.just.edu.cn (D.W.); 241110302101@stu.just.edu.cn (L.H.)

**Keywords:** underwater object detection, YOLOv11, object detection performance, ROV underwater robot

## Abstract

Despite the ocean’s vast energy reserves and extensive coverage of Earth’s surface, the complexity of the underwater environment has hindered effective target recognition. To mitigate the feature degradation caused by underwater scattering, wavelength-dependent absorption, and non-uniform illumination, this study proposes a degradation-aware YOLOv11s-based detection framework for underwater robotic object detection. The framework enhances detection robustness by improving feature reconstruction, channel–spatial attention, and bounding-box regression in a unified architecture. First, to address limitations in model parameters, spatial channel reconstruction convolutions (SCConv) replace certain traditional convolutions. Sequential reconstruction via SRU-CRU effectively suppresses spatial and channel redundancy, enabling a more precise capture of underwater target deformations and complex features. Second, the Shuffle Attention module enhances the interaction between channel and spatial features, improving the model’s fine-grained representation of underwater targets, highlighting granular objects and key textures. Finally, *Focaler-IoU* is employed to linearly remap the *IoU* interval, improving the accuracy and convergence stability of bounding-box regression. These components work together to improve the model’s robustness in degraded underwater scenes. In the underwater robotic detection task, the improved model achieves an mAP@0.5 of 88.4%, which is 3.2 percentage points higher than that of the baseline YOLOv11s. These results indicate that the proposed model improves detection accuracy while maintaining the real-time requirements of underwater robotic applications.

## 1. Introduction

The ocean contains abundant biological, mineral, and energy resources [1]. One of the most important steps toward sustainable maritime development and the preservation and improvement of the marine environment is establishing marine ranches. However, the techniques available for underwater photography are limited by the intrinsic features of the underwater environment [2]. Complex underwater environments, in contrast to terrestrial ones, pose several challenges, including uneven illumination, high turbidity, and stringent requirements for equipment pressure resistance and corrosion protection. Object detection is severely hampered by these differences in underwater imaging [3].

Unlike terrestrial photographs, underwater images often exhibit varying degrees of degradation, leading to blurred features, color distortion, and reduced contrast. These problems severely limit the effectiveness of underwater robots and their subsequent applications. To mitigate the decline in image quality under such specialized conditions, designing customized image processing methods to enhance image quality and improve practicality is crucial. Existing underwater image enhancement methods can be broadly divided into classical algorithms and deep learning-based methods.

Traditional techniques for improving underwater images primarily rely on mathematical models and artificial rules to compensate for issues such as light absorption, scattering, and color distortion caused by water. The goal is to improve the practical utility of underwater photos and ensure consistent performance in subsequent vision tasks by achieving accurate optical restoration. Histogram equalization (HE) [4], the Retinex algorithm [5], and the dark channel prior (DCP) [6] are classic techniques for improving conventional underwater images. Conventional image enhancement techniques are simple and can significantly improve the quality of underwater photos with minimal deterioration. These methods typically possess clear physical interpretability. However, they also exhibit the following shortcomings: they cannot eliminate noise or restore details in dimly lit environments with blue-green water haze; moreover, their physical parameters must be adjusted whenever environmental conditions change. Comprehensive performance improvements necessitate optimization or integration with other techniques. Unlike traditional image enhancement algorithms, deep learning-based methods enable neural networks to autonomously learn correlations among image feature mappings [7]. They may quickly learn pixel-level translation correlations using vast amounts of connected data rather than relying on manually constructed computational methods. Convolutional neural networks and generative adversarial networks are the two main deep learning techniques for image enhancement.

A technique based on combined local and global frequency-domain image processing was proposed by V. Voronin et al. [8]. The fundamental notion is applying a logarithmic transform, histogram matching, and a spatial equalization technique to various image blocks. A. Saleem et al. [9] outlined the most popular models and offered a comparative analysis of their performance at various levels. The findings demonstrate that the chosen image enhancement models can generate much higher-quality images, with certain models outperforming others at specific depths. This technique restores equilibrium across color-based channels, employs a simple Transformer to separate absorption and scattering bands, incorporates deformable convolutional networks to detect blurry and unseen regions automatically, and applies robust protection against edge blurring caused by global augmentation via scattering adjustment at extremely turbid locations. To accomplish feature extraction and reconstruction, Wang et al. [10] integrated a convolutional network with a U-shaped structure to create an underwater generative adversarial network (GAN). This model enhances the material description of underwater light movement through adversarial training in an unsupervised setting, resulting in significant improvements in color-space restoration performance and image visual realism.

Considering the degraded visual conditions in underwater scenes, this study focuses on improving the feature representation and localization ability of object detection models rather than directly restoring underwater images.

Object detection is an important task in computer vision and has received increasing attention in recent years [11]. Two-stage detectors first generate region proposals and then perform classification and bounding-box regression. Although Fast R-CNN [12] is a representative two-stage method with high detection accuracy, its relatively slow inference speed limits its use in real-time systems, making it unsuitable for autonomous ROVs operating in dynamic underwater environments. Single-stage detectors directly predict object categories and bounding boxes in an end-to-end manner, making them more suitable for real-time underwater robotic applications. Representative single-stage detectors include SSD [13], YOLO [14], and FCOS [15], which provide a favorable balance between detection accuracy and inference speed. However, these generic detectors still struggle with underwater-specific degradations, such as low contrast, color distortion, and blurred object boundaries, leading to missed detections in complex underwater scenes. In practical underwater environments, object detection is highly sensitive to illumination changes. Images captured by underwater robots often suffer from blurred targets and low contrast, which challenges the adaptability of detection models.

Zhong et al. [16] proposed CAL-SSD, a compact image identification algorithm that uses a synchronized concentration approach. According to experimental findings, CAL-SSD achieves the best possible balance between *precision* and speed while drastically reducing model complexity and parameter count. To achieve high accuracy, Archana V et al. [17] presented state-of-the-art object detection approaches that include many components and algorithms for deep learning, detection algorithms, datasets, and the software/hardware required for object identification. There is a discussion of benchmark datasets for object detection. The COCO benchmark dataset is used to develop the proposed technique, which combines CNNs with RetinaNet. Zhao et al. [18] proposed an enhanced Faster R-CNN-based target detection algorithm (iFaster R-CNN). According to experimental findings, iFaster RCNN detects underwater fish more accurately. Hussain et al. [19] employed a systematic methodology to trace the evolutionary trajectory of YOLO variants. By dissecting the internal architectural composition of each variant, he conducts an in-depth analysis of its structural components. Subsequently, he highlights the key architectural innovations introduced by each variant, reveals the incremental optimization pathways, and incorporates benchmark performance metrics to provide quantitative assessments of their capabilities. Further demonstrations showcase the practical performance of YOLO variants across diverse domains, highlighting their real-world application value. This structured approach ensures a comprehensive examination of YOLO’s evolutionary journey, systematically elucidating its internal technical progression and benchmarking performance before delving into domain-specific applications. By leveraging multi-edge device collaborative training, the performance of YOLO models is enhanced while simultaneously strengthening privacy protection, adaptability, and generalization capabilities. YOLO, on the other hand, has a clear advantage in this detection area. The YOLO series has steadily become a top competitor in underwater object detection, driven by the ongoing development of single-stage algorithms that offer improved accuracy and faster response times.

Wang et al. [20] proposed a YOLOv3-based underwater object detection technique. Subsequently, Huang et al. [21] introduced DPT-YOLO, a novel network architecture that enhances the traditional YOLO framework with a dual-path transformer module, improving geometric feature extraction for target detection. They further proposed a category-weighted YOLO network that improved underwater object detection *precision* by using a category-weighted loss function and incorporating adaptive dimensions.

In practical applications, underwater target detection is significantly more challenging due to the diversity and complexity of the underwater environment. This often results in poor image quality captured by underwater robots and reduced target clarity. Wu Reddy et al. [22] assessed the mechanical identification performance of three cutting-edge deep learning-based object detection models: YOLOv11, Faster R-CNN, and RetinaNet. They discovered that YOLOv11 strikes the optimum compromise between real-time execution and detection accuracy. In the same year, the performance of YOLOv8m, YOLOv8l, YOLOv11m, and YOLOv11l is thoroughly assessed by Selcuk et al. [23] in comparison to the widely used Faster R-CNN model in the literature for DRF detection. The findings indicate that YOLOv11l achieved the maximum *precision* and delivered the best performance on the validation set. YOLOv11 provides extremely effective feature extraction, successfully tackling issues such as complex backgrounds, small target sizes, and low underwater levels of detail. Later iterations of the YOLO series are better suited to GPU-optimized environments or high-throughput, real-time inference (such as video processing). However, for underwater object detection, YOLOv11 remains the more robust choice. Consequently, many researchers have focused on improving YOLOv11 as their primary target. Hong et al. [24] proposed the improved YOLO-HC model based on the YOLOv11 framework. They constructed a Multi-Scale Dilated Attention Module (MSDA_C2PSA) to enhance cross-scale feature extraction, employed a bidirectional weighted feature pyramid to optimize multi-level object representation fusion, and further improved detection robustness by integrating multiple attention mechanisms via a dynamic detection head. To improve recognition effectiveness in aquatic situations, Chen et al. [25] suggested an underwater object detection model that incorporates a multi-scale attentiveness method.

The model significantly improved its ability to recover edge and texture details from underwater photos by replacing the first two convolutional layers of the YOLOv11 backbone with the Laplace–Gaussian stem (LoGStem). Chang et al. [26] proposed a lightweight detection algorithm, YOLOv11-MSG. This method uses the lightweight MobileNetV3 architecture instead of the traditional YOLOv11s backbone. It further reduces computational complexity by substituting the original SPPF with Sim-SPPF and incorporating the GAM global attention mechanism, making it more suitable for embedded devices. Gu et al. [27] first embedded the Faster-Block module into the YOLOv11 backbone to reduce parameter count while improving feature extraction. Subsequently, they replaced it with Slim-Neck to lower computational complexity without sacrificing accuracy. For small-object detection, they added a Triplet-Attention module to detect minute defects. Kıratlı et al. [28] proposed YOLOv11-EFAC, using YOLOv11n as its baseline, and employed a multi-level optimization strategy. The lightweight, high-quality feature extraction enabled by the efficient Net-B0 backbone makes YOLOv11-EFAC a reliable, real-time solution for vital drone applications when resources are limited.

Despite these recent advancements, a significant gap remains between theoretical laboratory performance and practical underwater robotic deployment. While recent specialized methods (e.g., DPT-YOLO and LGM-YOLOv11) have successfully improved detection accuracy through complex feature-refining paths, their high computational latency often precludes real-time execution on mobile ROVs with stringent energy budgets. Moreover, many existing frameworks rely on a simple ‘plug-and-play’ assembly of off-the-shelf modules, but they often do not explicitly link underwater degradation characteristics with network-level design.

To address these challenges, this study proposes a degradation-aware YOLOv11s-based detection framework for underwater robotic object detection. The proposed framework is not designed as a simple combination of independent modules. Instead, it is developed according to the feature-level effects of underwater image degradation. In underwater scenes, scattering and turbidity often introduce redundant background responses; wavelength-dependent absorption weakens the discriminative information of color-related channels; non-uniform illumination causes unstable spatial activations; and blurred object boundaries make bounding-box regression difficult, especially for small and camouflaged targets. Therefore, this work establishes a unified framework that jointly enhances feature reconstruction, channel–spatial interaction, and localization accuracy. The main contributions of this work are summarized as follows:

(1) A degradation-aware feature reconstruction strategy is introduced by replacing selected standard convolutions in YOLOv11s with SCConv. The SRU and CRU components suppress redundant spatial and channel responses caused by underwater turbidity, scattering, and color attenuation, thereby improving the representation of degraded underwater targets.
(2) A lightweight Shuffle Attention module is incorporated to strengthen channel–spatial feature interaction. This design helps the network focus on informative target regions and fine-grained texture cues under non-uniform illumination and complex underwater backgrounds.
(3) *Focaler-IoU* is adopted to improve bounding-box regression for underwater targets with blurred boundaries, small scales, and partial occlusion. By remapping the *IoU* interval, the regression process can better adapt to difficult underwater samples.
(4) Extensive experiments, including ablation studies, generalization evaluation, challenging-scenario analysis, and underwater robotic tests, are conducted to verify the effectiveness and practical applicability of the proposed framework.

## 2. Materials and Methods

### 2.1. Overall Framework of the ROV

The robotic body is equipped with fundamental modules such as a manipulator. Furthermore, to meet research requirements for underwater target detection, a custom-designed 1080P camera (assembled at Jiangsu University of Science and Technology, Zhenjiang, China) was installed on the ROV to transmit underwater imagery to external systems.

**Figure 1:**
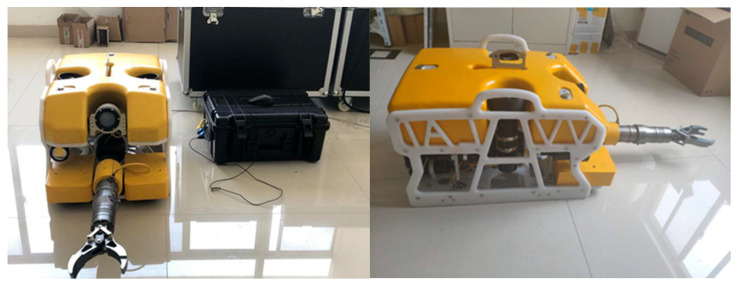
Physical image of the ROV underwater robot.

The surface control console was equipped with an NVIDIA GeForce RTX 4070 Ti graphics processing unit (NVIDIA Corporation, Santa Clara, CA, USA). This configuration is designed to meet the high computational demands of deep learning algorithms, ensuring real-time performance and accuracy in target detection. Additionally, the software system running on the console efficiently processes video data from the underwater robot, enabling seamless execution of functions such as target detection, recognition, and tracking. It has been specifically optimized for robustness in complex marine engineering applications. A 20 kg-class ROV system was designed and assembled in our laboratory at Jiangsu University of Science and Technology, Zhenjiang, China. The system adopts a modular architecture and is composed primarily of two major components: the robotic body and the surface control console. The tethered ROV (Remotely Operated Vehicle) is illustrated in Figure 1.

**Figure 2:**
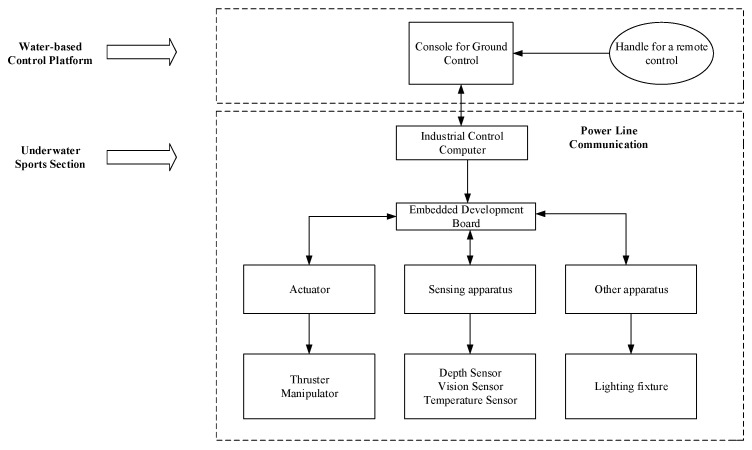
ROV structural diagram.

The ROV system primarily consists of an underwater operating unit and a surface monitoring platform, physically connected via a composite cable and communicating via power-line communication. This architectural design not only ensures reliable energy supply and information transmission but also optimizes the efficiency of human–machine interaction, demonstrating significant advantages in scientific tasks such as underwater environmental monitoring and biological sample acquisition. The surface monitoring platform integrates a multifunctional joystick, a high-resolution monitor, and standard input devices to form a comprehensive control interface. This enables operators to execute omnidirectional motion commands while simultaneously visualizing real-time equipment status. The structure of the tethered underwater robotic system is illustrated in Figure 2.

**Figure 3:**
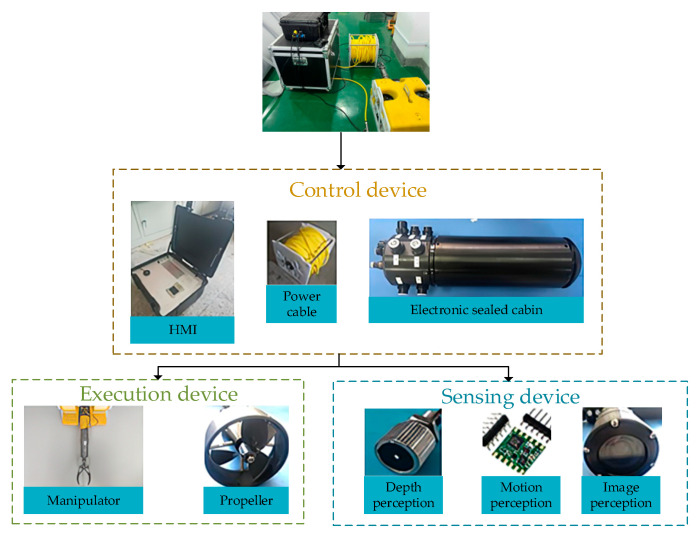
Module structure diagram.

The control unit is responsible for transmitting control signals and receiving feedback signals, ensuring steady information transfer between terrestrial and underwater environments. The ROV’s operating environment under the sea informs its decision-making. The execution unit manages the ROV’s propulsion and adjusts the motion posture to match the current water flow. The sensing unit perceives the operational environment, collects underwater data, and reports operational status while ensuring the proper functioning of cameras, lighting equipment, and other peripheral devices. The diagram is shown in Figure 3.

### 2.2. Research on Object Detection Algorithms Based on YOLOv11

**Figure 4:**
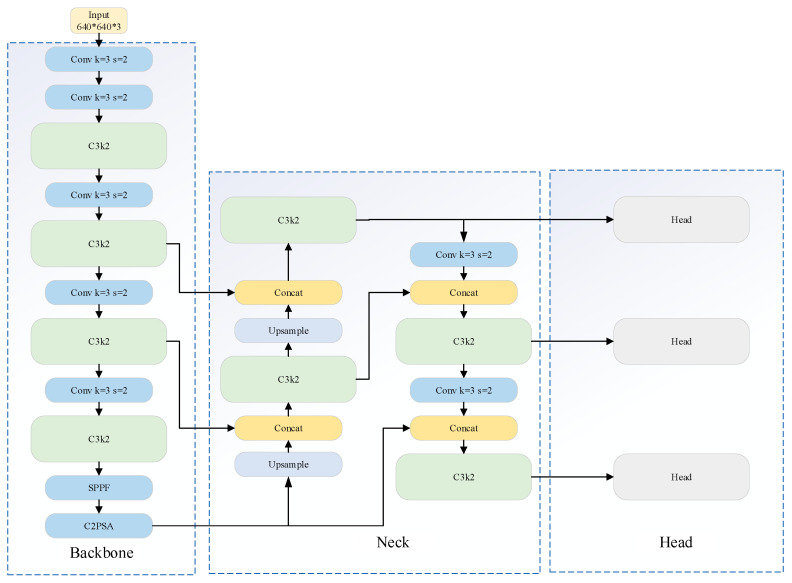
YOLOv11 architecture diagram. Different colors indicate different functional modules, including convolutional blocks, feature fusion layers, and detection heads.

The YOLOv11 series represents the most advanced, lightweight, and efficient models within the YOLO algorithm family. As illustrated in Figure 4, YOLOv11’s architecture comprises three components: Backbone, Neck, and Head. Equipped with an ultra-lightweight model, YOLOv11 operates faster and more efficiently than previous YOLO iterations, enabling it to handle a broader range of computer vision tasks. To accommodate varying performance and resource requirements, YOLOv11 offers five distinct models: YOLOv11n, YOLOv11s, YOLOv11m, YOLOv11l, and YOLOv11x. This paper selects the YOLOv11s model, which demonstrates significant improvements in speed, accuracy, and adaptability. It is an efficient, flexible, and powerful object detection model suitable for a wide range of practical applications. Figure 4 presents a schematic diagram of this network architecture.

**Figure 5:**
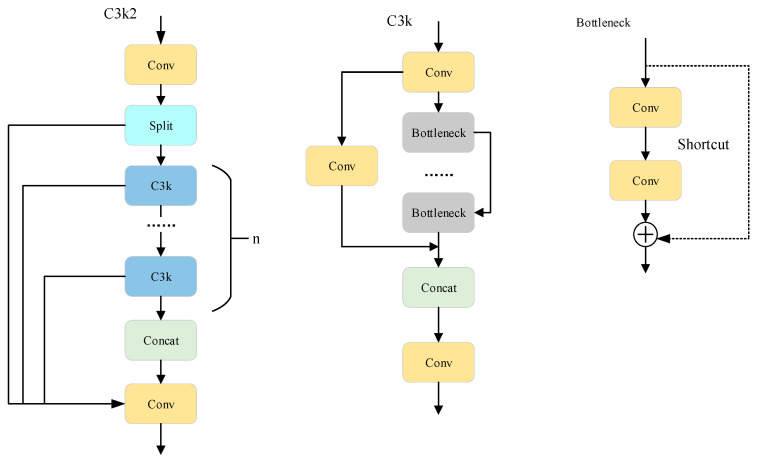
C3K2 structural diagram. Different colors represent convolution, split, bottleneck, concatenation, and shortcut operations.

To minimize network size and improve performance, the backbone employs a sequence of convolutional and deconvolutional layers with residual connections and bottleneck structures for feature extraction. YOLOv11 uses C3K2 blocks to extract features across different backbone phases. While preserving the model’s ability to extract crucial information from images, smaller 3 × 3 kernels enable more efficient computation. The C3K2 block, a development of the CSP bottleneck first seen in previous iterations, is the central component of the YOLOv11 backbone. The C3K2 module divides feature maps and applies a sequence of smaller (3 × 3) convolutions to maximize information flow throughout the network. Compared to larger convolutions, this method is quicker and requires less computing power. The C3K2 module uses fewer parameters than YOLOv8s C2f module and enhances feature representation by processing smaller, independent feature maps and merging them after several convolutions. Its structure is illustrated in Figure 5.

**Figure 6:**
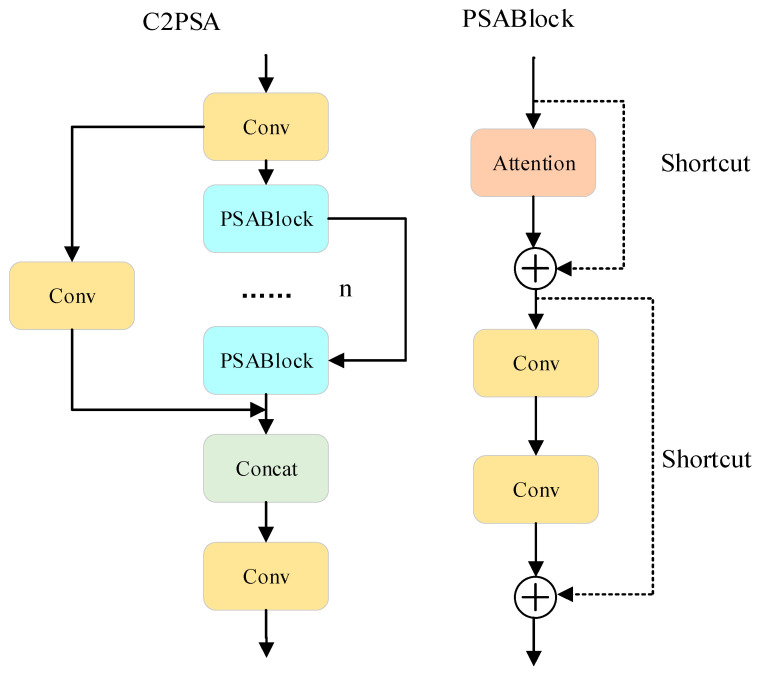
C2PSA structural diagram. Different colors represent convolution, split, bottleneck, concatenation, and shortcut operations.

The C2PSA block employs two PSA (Partial Spatial Attention) modules that operate on different branches of the feature map and then concatenate the outputs, similar to the C2F block structure. This configuration ensures the model focuses on spatial information while balancing computational cost and detection accuracy. The C2PSA module refines the model’s ability to selectively focus on regions of interest by applying spatial attention to extracted features, as illustrated in its structural diagram in Figure 6.

The neck network is located between the backbone and the detection head. It fuses multi-scale features extracted by the backbone and provides enriched representations for final prediction, enabling the model to detect underwater objects at different scales. The detection head predicts object categories, bounding-box locations, and confidence scores based on the fused feature maps. As the final prediction component, the detection head directly affects both detection accuracy and inference efficiency.

### 2.3. Improvements to the YOLOv11s Object Detection Network

Underwater image degradation primarily affects object detection through feature redundancy, unstable attention responses, and localization difficulties. Specifically, scattering, turbidity, and wavelength-dependent absorption tend to introduce redundant spatial and channel responses, which weaken target contours and discriminative features. Non-uniform illumination and complex backgrounds further disturb the network’s attention to informative target regions. In addition, blurred boundaries, small target scales, and partial occlusion make bounding-box regression less stable. Therefore, the proposed framework introduces SCConv, Shuffle Attention, and *Focaler-IoU* in a degradation-aware manner. SCConv is used to reconstruct spatial and channel features, while Shuffle Attention strengthens channel–spatial interaction, and *Focaler-IoU* improves the localization of difficult underwater samples. In this way, the three components form a complementary design rather than a simple stacking of independent modules.

#### 2.3.1. Improvements to the Original Convolution

Based on the degradation-aware design described above, selected standard convolutions in YOLOv11s are replaced with SCConv. In degraded underwater scenes, redundant spatial and channel responses can obscure target contours and weaken discriminative features. SCConv is therefore introduced to suppress such redundancy and improve the representation of underwater targets. SCConv has demonstrated potential for tasks such as image classification and detection, improving accuracy while reducing redundancy and computational cost. It can be seamlessly plugged in to replace standard convolutions.

**Figure 7:**
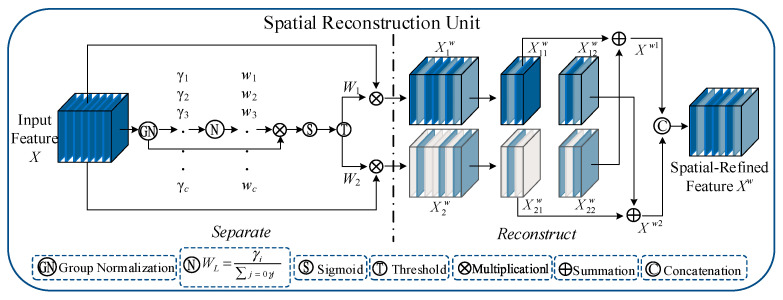
SRU schematic diagram.

SCConv consists of two units: the Spatial Reconstruction Unit (SRU) and the Channel Reconstruction Unit (CRU). The SRU leverages separation and reconstruction operations to extract spatial redundancy from features. Specifically, it evaluates the information content of different feature maps through the scaling factor of the Group Normalization (GN) layer. Feature maps are then partitioned into information-rich and information-poor regions based on their weights. These two portions are subsequently combined via a cross-reconstruction operation to reduce spatial redundancy and enhance feature representation, as illustrated in Figure 7.

**Figure 8:**
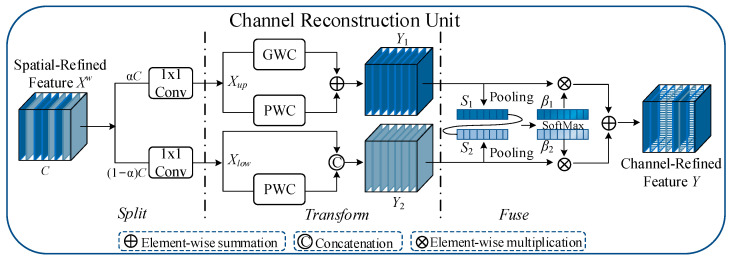
CRU schematic diagram.

CRU employs a split–transform–fuse strategy to extract channel redundancy from features. First, the channels of spatially refined feature maps are split and compressed. Then, efficient convolutional operations (such as GWC and PWC) transform the split feature maps to extract high-level representative information while reducing computational costs. Finally, a simplified SKNet method adaptively fuses the output features, thereby reducing channel-dimension redundancy, as shown in Figure 8.

SCConv is a plug-and-play module that can directly replace standard convolutions in various convolutional neural networks without requiring additional modifications to the model architecture. The design of SRU and CRU enhances feature representation capabilities, generating more representative and expressive features. By exploiting redundancy in spatial and channel dimensions, SCConv reduces computational complexity and parameter count, thereby lowering computational costs. Experimental results demonstrate that models incorporating SCConv achieve superior performance while reducing complexity and computational costs, outperforming other state-of-the-art methods in tasks such as image classification and object detection.

#### 2.3.2. Improvements to Attention Mechanisms

To further enhance YOLOv11s feature representation capabilities in complex underwater scenes, this paper introduces Shuffle Attention (SA), a lightweight yet effective channel–spatial hybrid attention module. By grouping, rearranging, and multi-branch aggregating, the network can more precisely focus on regions and channel features meaningful for object detection.

For a given feature map $X\in R^{C\times H\times W}$, *X* is first split into *G* groupings along the distribution length by SA. The formula is as follows:

(1) $$X=\left[X_{1},\ldots X_{G}\right],X_{k}\in R^{\frac{C}{G}\times H\times W}$$

At the start of each attention unit, the input is split along the channel dimension into two branches: $X_{k1}$ and $X_{k2}$. While one branch uses inter-channel connections to generate a channel attention map, the other branch uses spatial relationships among features to generate a spatial attention map. This dual approach enables the model to focus on both “what” and “where” are meaningful.

To fully capture inter-channel dependencies, SA employs Global Average Pooling (GAP) to generate channel-level statistics. The formula is as follows:

(2) $$s=F_{gp}(X_{k1})=\frac{1}{H\times W}\sum_{i=1}^{H}\sum_{j=1}^{W}X_{k=1}(i,j)$$

Subsequently, a compact feature is produced using a simple gating mechanism with sigmoid activation, enabling precise, adaptive selection guidance. The final output of the channel attention is obtained as follows:

(3) $$X_{k1}^{\prime }=\sigma (F(s))\cdot X_{k1}=\sigma (W_{1}s+b_{1})\cdot X_{k1}$$

Unlike channel attention, spatial attention focuses on identifying “where” information-rich regions lie, thereby complementing it. First, the input undergoes GroupNorm processing to extract spatial-level statistics, followed by representation enhancement ${\overset{\wedge }{X}}_{k2}$. The following is the equation:

(4) $$X_{k2}^{\prime }=\sigma (W_{2}\cdot GN(X_{k2})+b_{2})\cdot X_{k2}$$

**Figure 9:**
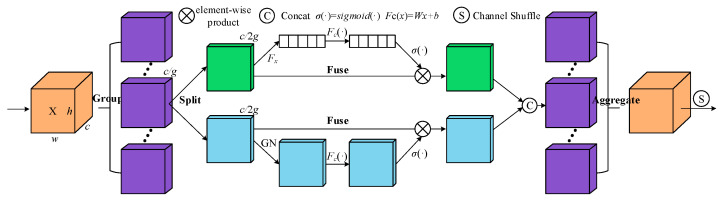
Shuffle attention architecture diagram. Different colors indicate different feature branches and operations in the channel and spatial attention paths.

Subsequently, all sub-features are aggregated. Finally, similar to ShuffleNet v2, a “channel shuffle” operation is employed to facilitate cross-channel information flow, as illustrated in Figure 9.

#### 2.3.3. Improvements to the Loss Function

**Figure 10:**
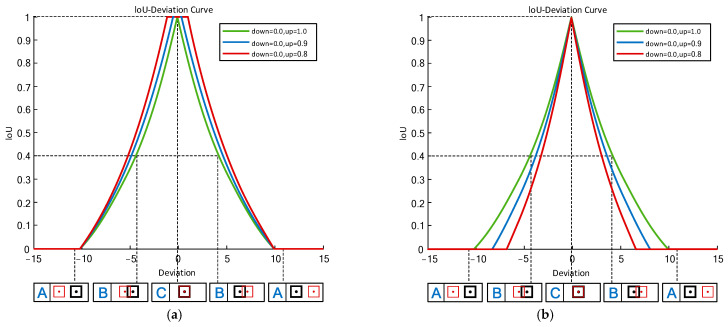
Linear interval mapping curves for different sample types: (**a**) difficult samples; (**b**) easy samples. Here, A, B, and C denote zero, partial, and complete overlap states between the ground truth (solid black) and predicted (solid red) boxes. Vertical dashed lines align the curve values with these spatial states.

Sample imbalance is common in object detection. According to localization difficulty, training samples can be roughly divided into easy and hard samples. From the target scale perspective, general detection targets can be regarded as easy samples, whereas extremely small targets, due to the difficulty of precise localization, can be considered difficult samples. For detection tasks dominated by easy samples, focusing bounding-box regression on them will improve detection performance. Conversely, for tasks with a high proportion of hard samples, the bounding-box regression should focus on the hard samples, as illustrated in Figure 10.

To focus on different regression samples across various detection tasks, we employ linear interval mapping to reconstruct the *IoU* loss, thereby enhancing bounding-box regression performance. The formula is as follows:

(5) $$IoU^{focaler}=\left\{\begin{array}{l} 0,IoU<d\\ \frac{IoU-d}{u-d},d\ll IoU\ll u\\ 1,IoU>u \end{array}\right.$$

$IoU^{focaler}$ denotes the reconstructed *Focaler-IoU*, where *IoU* represents the original *IoU* value, with $\left[d,u\right]\in \left[0,1\right]$. By adjusting the values of *d* and *u*, one $IoU^{focaler}$ can focus on different regression samples.

The loss is defined as follows:

(6) $$L_{Focaler-IoU}=1-IoU^{focaler}$$

When *Focaler-IoU* is combined with existing *IoU*-based bounding-box regression losses, $L_{Focaler-GIoU}$, $L_{Focaler-DIoU}$, $L_{Focaler-CIoU}$, $L_{Focaler-EIoU}$, and $L_{Focaler-SIoU}$ are defined as follows:

(7) $$\begin{array}{l} L_{Focaler-GIoU}=L_{GIoU}+IoU-IoU^{Focaler}\\ L_{Focaler-DIoU}=L_{DIoU}+IoU-IoU^{Focaler}\\ L_{Focaler-CIoU}=L_{CIoU}+IoU-IoU^{Focaler}\\ L_{Focaler-EIoU}=L_{EIoU}+IoU-IoU^{Focaler}\\ L_{Focaler-SIoU}=L_{SIoU}+IoU-IoU^{Focaler} \end{array}$$

Bounding-box regression plays a pivotal role in object detection, where localization accuracy largely depends on the regression loss function. Existing research enhances regression performance by leveraging geometric relationships among boxes, yet it overlooks the impact of the distribution of samples from easy to difficult on regression. This paper introduces the *Focaler-IoU* method, which enhances detector performance across diverse detection tasks by focusing on different regression samples. It achieves this focus on easy and hard samples through linear interval mapping that reconstructs the original *IoU* loss.

## 3. Results

### 3.1. Data Collection and Experimental Setup

**Figure 11:**
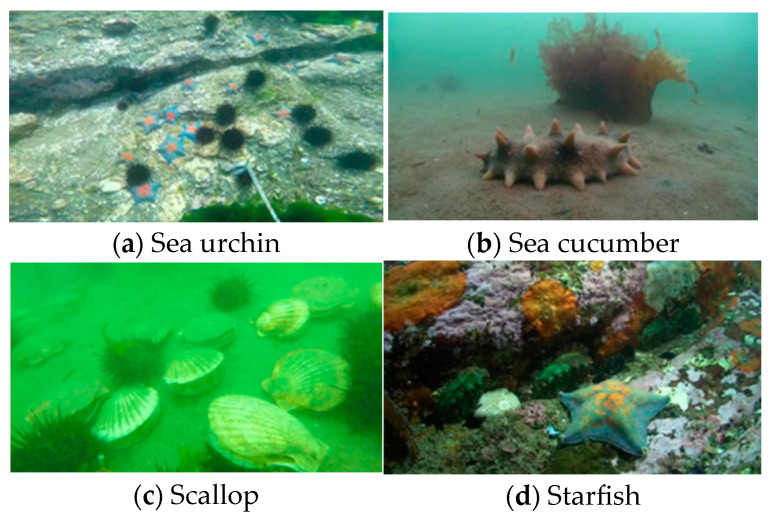
Images from the dataset.

The URPC dataset is used in this investigation, as shown in Figure 11. To create an enlarged dataset of 5543 underwater target photos, we gathered and selected photos of sea urchins, sea cucumbers, scallops, and starfish from internet sources. To promote underwater vision technology for uses such as underwater object identification, recognition, and classification, the Underwater Robotics Professional Competition organization has been releasing the URPC dataset since 2017.

**Figure 12:**
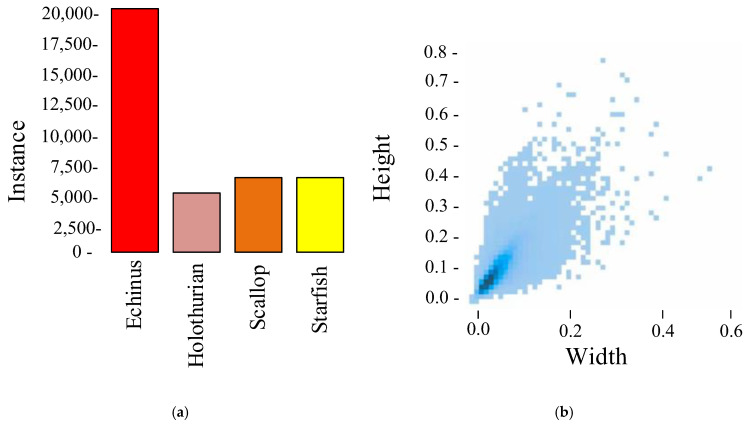
Data distribution of the URPC dataset: (**a**) number of instances in each object category; (**b**) normalized bounding-box width and height distribution. Different colors in (**a**) indicate different object categories.

As seen in Figure 12a, this dataset includes items from many categories. The distribution of bounding-box dimensions is shown in Figure 12b, which demonstrates that the majority of objects are rather small, with sizes mostly falling within 0.0 and 0.2. The data is divided into training and validation sets at a 9:1 ratio to enable efficient learning by the designated model on this dataset.

To evaluate the generalization ability of the proposed method, an additional experiment was conducted using the publicly available RUOD dataset. The RUOD dataset contains 13,950 high-resolution images covering ten underwater object categories, including sea cucumbers, sea urchins, scallops, starfish, corals, and divers. To keep the experimental protocol consistent with the URPC experiments, the RUOD dataset was randomly divided into training and validation subsets at a ratio of 9:1, with 12,555 images for training and 1395 images for validation. All compared models were trained and evaluated using the same data split to ensure a fair comparison. The dataset partitions used in this study are summarized in Table 1.

The hyperparameters were first set according to the empirical defaults of YOLOv11 and then adjusted through iterative experiments on the underwater datasets. As listed in Table 2, the batch size was set to 4 due to the 12-GB GPU memory limit and the need to maintain high-resolution inputs for small-target detection. A hold-out validation strategy was used in all experiments. The maximum number of training epochs was set to 100. Early stopping was applied when the validation performance no longer improved, and the checkpoint with the best validation performance was used for the final evaluation.

### 3.2. Evaluation Indicators

The performance of object detection algorithms is thoroughly assessed in this work utilizing average precision (*AP*), mean average precision (*mAP*), and frames per second (FPS).

*Precision* measures the proportion of correctly detected positive samples among all samples predicted as positive. The following is the equation:

(8) $$\mathrm{Pr}ecision=\frac{TP}{TP+FP}$$

*Recall* measures the proportion of correctly detected positive samples among all ground-truth positive samples. The following is the equation:

(9) Recall=TPTP+FN

The *F*_1_ score is the harmonic mean of *precision* and *recall* and provides a balanced evaluation of detection performance. The following formula is used to determine the *F*_1_ score:

(10) $$F_{1}=\frac{2\times precision\times recall}{precision+recall}=\frac{2TP}{2TP+FP+FN}$$

*AP* is calculated as the area under the *precision*–*recall* curve for each object category. The following is the equation:

(11) $$AP=\int_{0}^{1}PdR$$

Here, the *recall* rate is denoted as *R*.

The average of all category *AP* scores is used to calculate *mAP*, a composite statistic that integrates *recall* and *precision*. mAP@0.5 is employed to evaluate the *precision* of popular mAP@0.5 models. The following is the expression for the equation:

(12) $$mAP=\frac{\sum_{i=1}^{N}AP_{i}}{N}$$

### 3.3. Experimental Results and Analysis

#### 3.3.1. Experiments on the URPC Dataset

**Figure 13:**
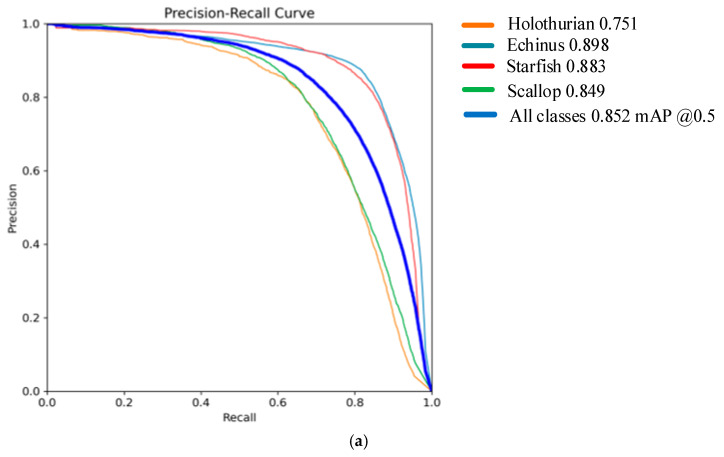

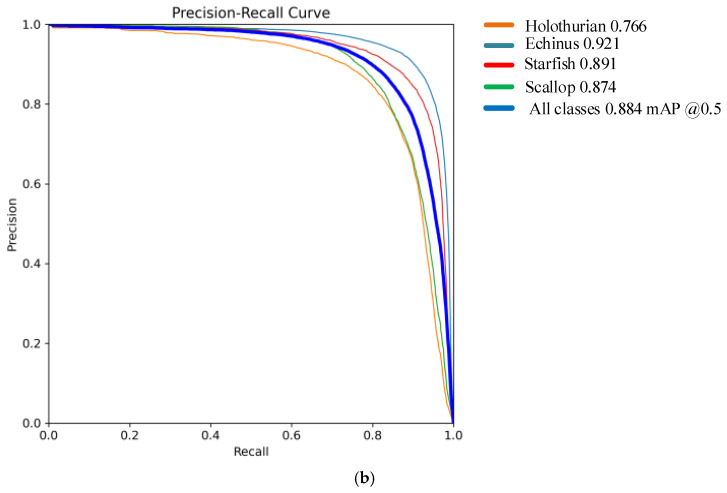
*Precision*–*recall* curves of the baseline YOLOv11s and the improved YOLOv11s on the URPC dataset: (**a**) baseline YOLOv11s; (**b**) improved YOLOv11s.

Figure 13 shows the *precision*–*recall* curves of the baseline YOLOv11s and the improved YOLOv11s on the URPC dataset. As shown in the figure, the improved model achieves an mAP@0.5 of 88.4%, representing a 3.2% increase over the original model. Among the classes, sea urchins exhibit distinct color features, achieving a high AP of 92.1%, whereas sea cucumbers, whose color closely resembles the background, attain a lower *AP* of 76.6%.

**Figure 14:**
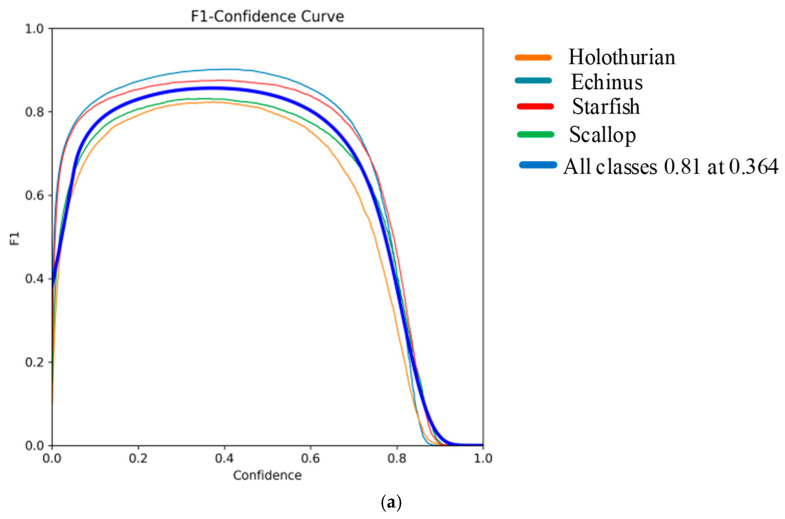

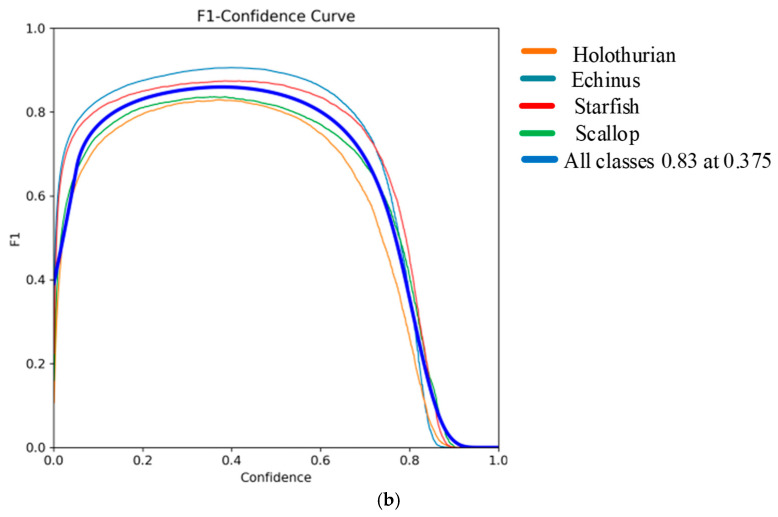
*F*_1_-confidence curves of the baseline YOLOv11s and the improved YOLOv11s on the URPC dataset: (**a**) baseline YOLOv11s; (**b**) improved YOLOv11s.

Figure 14 shows the *F*_1_ score curves for the models before and after improvement. Similarly, the improved model outperforms the original.

**Figure 15:**
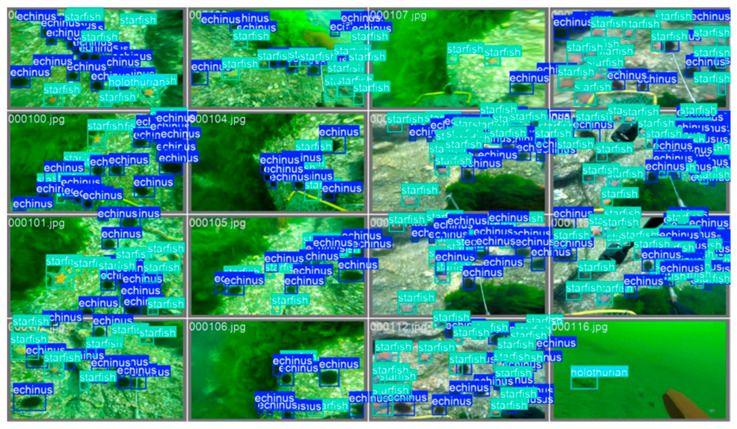
Representative detection results of the improved YOLOv11s on the URPC validation set. Colored bounding boxes indicate detected object categories, and the labels show the predicted class names and confidence scores.

Figure 15 shows representative detection results of the improved model on the validation set. The results indicate that the improved model can detect small and partially camouflaged underwater targets more effectively, demonstrating enhanced feature extraction capability in complex underwater scenes.

**Figure 16:**
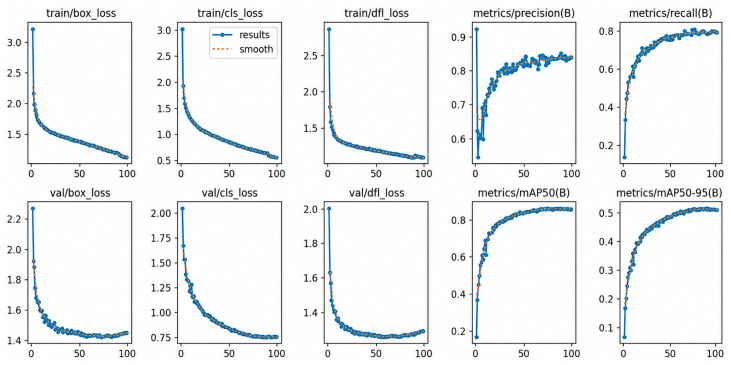
Training and validation curves of the improved model on the URPC dataset.

Figure 16 shows the updated training and validation curves of the improved model on the URPC dataset. The box loss, classification loss, and DFL loss decrease steadily during training, while *precision*, *recall*, mAP@0.5, and mAP@0.5:0.95 gradually increase and become relatively stable in the later epochs. These results indicate that the model reaches a satisfactory training state within 100 epochs. The final evaluation was conducted using the checkpoint with the best validation performance.

#### 3.3.2. Experiments on the RUOD Dataset

To verify that the performance gains of the improved YOLOv11s are not limited to a specific dataset, a generalization experiment was conducted on the RUOD dataset using the fixed 9:1 split described in Section 3.1. All models were trained and evaluated under the same experimental settings, including the same input resolution, optimizer, batch size, learning rate, confidence threshold, and hardware platform.

**Figure 17:**
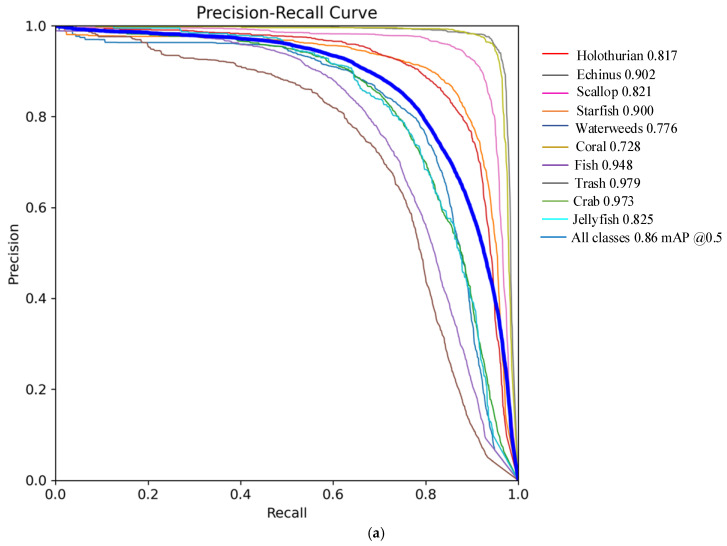

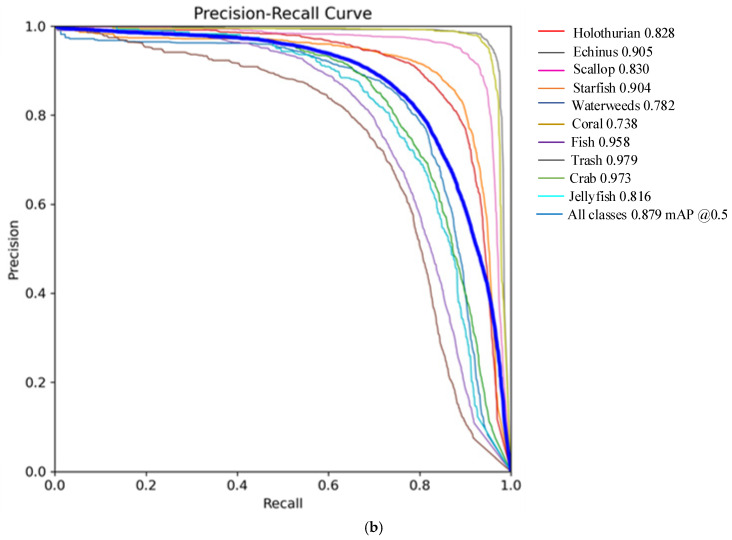
*Precision*–*recall* curves of the baseline and improved models on the RUOD dataset: (**a**) baseline YOLOv11s; (**b**) improved YOLOv11s. Each colored curve represents the *precision*–*recall* result of one object category, and the bold blue curve represents the average result across all classes.

As shown in Figure 17, the baseline YOLOv11s achieved an mAP@0.5 of 86.0%, while our improved model reached 87.9%, representing an absolute improvement of 1.9%. This improvement on a different underwater dataset suggests that the proposed components can enhance the model’s generalization ability under degraded underwater imaging conditions.

**Figure 18:**
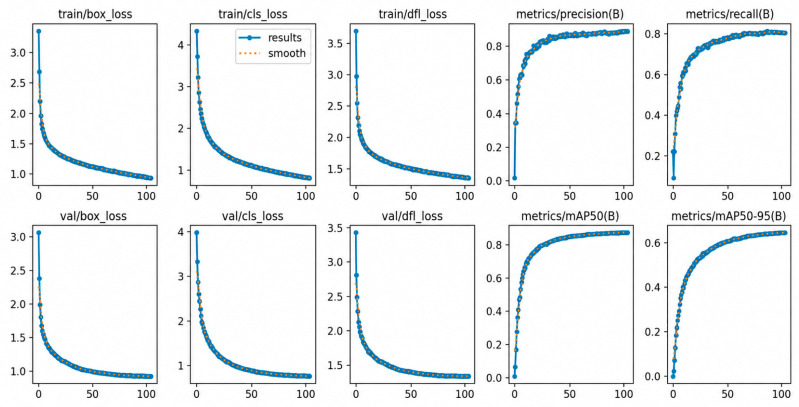
Training and validation curves of the improved model on the RUOD dataset.

An analysis of the training process reveals that the bounding-box loss, classification loss, and distribution-focused loss all converge well during both training and validation. The model demonstrates rapid improvements in *precision*, *recall*, and average precision. Figure 18 presents the updated training and validation curves of the improved model on the RUOD dataset. The loss curves show a clear decreasing trend during training, while *precision*, *recall*, mAP@0.5, and mAP@0.5:0.95 gradually increase and remain within a relatively stable range in the later epochs. These results suggest that the model reaches a satisfactory training state within 100 epochs. The final reported results are based on the checkpoint with the best validation performance rather than the last training epoch.

#### 3.3.3. Evaluation of a Subset of Challenging Underwater Scenarios

To further analyze whether the proposed components address specific underwater detection challenges, the validation set was manually divided into three challenging subsets, including turbid and color-cast scenes, camouflaged target scenes, and clustered small-object scenes.

**Figure 19:**
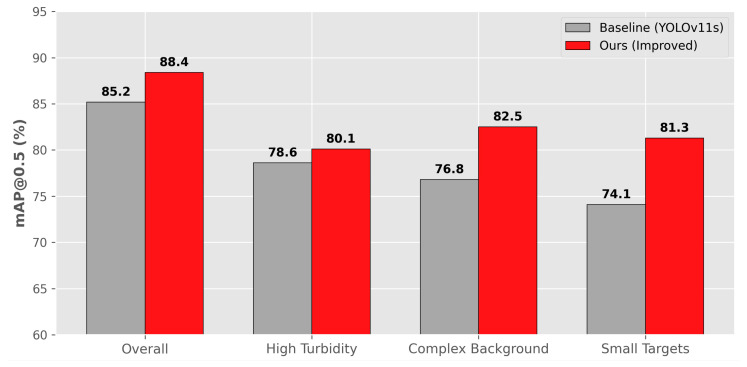
Detection performance comparison across different underwater scenarios.

As illustrated in Figure 19, in highly turbid, color-cast environments where the baseline model struggles, our approach achieved a 3.2% improvement in *mAP*. This result indicates that SCConv can help suppress spatial and channel redundancies caused by severe underwater light scattering. For highly camouflaged targets that blend into cluttered rocky seabeds (e.g., sea cucumbers), the model yielded a 1.5% *mAP* increase, suggesting that Shuffle Attention helps highlight discriminative texture and region cues under complex backgrounds. Additionally, in scenarios dominated by clustered or distant small objects, a significant 7.2% *mAP* gain was observed, showing that *Focaler-IoU* improves bounding-box regression for difficult underwater samples.

The above results further support the degradation-aware design of the proposed framework. The improvement in turbid and color-cast scenes indicates that SCConv can reduce redundant spatial and channel responses caused by underwater scattering and wavelength-dependent absorption. The gain in camouflaged scenes suggests that Shuffle Attention helps the detector focus on discriminative texture and region cues under complex backgrounds. The larger improvement in clustered and small-object scenes shows that *Focaler-IoU* is beneficial for difficult bounding-box regression. These findings demonstrate that the three components contribute to different degradation-induced detection difficulties and work in a complementary manner, rather than forming a simple module-stacking strategy.

#### 3.3.4. Comparison with Other Models on the URPC and RUOD Datasets

Comparative experiments were conducted on the URPC and RUOD datasets to assess the performance and generalization ability of the proposed method. The compared models include the classical detector SSD, recent YOLO-based models, RT-DETR-R18, and two underwater-oriented methods, namely DPT-YOLO and LGM-YOLOv11. All models were trained and evaluated using the same dataset splits and experimental settings to ensure a fair comparison.

Table 3 reports the comparison results on the URPC dataset. The improved YOLOv11s achieves the highest mAP@0.5 of 88.4%, which is 3.2 percentage points higher than that of the baseline YOLOv11s. It also outperforms recent detectors such as YOLOv8s, YOLOv9s, YOLOv10s, RT-DETR-R18, and YOLOv12s in overall detection accuracy. Although its inference speed is slightly lower than that of some lightweight YOLO-based models, it still reaches 87 FPS, satisfying the real-time requirement of underwater robotic detection. These results indicate that the proposed framework provides a favorable balance between detection accuracy and inference efficiency on the main underwater robotic object detection task.

The generalization performance was further evaluated on the RUOD dataset, and the results are summarized in Table 4. The improved YOLOv11s achieves 87.9% mAP@0.5, exceeding the baseline YOLOv11s by 1.9 percentage points. Compared with other recent detectors, the proposed method obtains the best overall detection accuracy while maintaining real-time inference performance. This improvement on a different underwater dataset suggests that the proposed framework has good generalization ability under varied underwater imaging conditions.

To evaluate the stability of the experimental results, three independent training runs were conducted for the baseline YOLOv11s and the improved YOLOv11s on both datasets. Different random seeds were used in each run, while the dataset splits, input resolution, hyperparameters, training epochs, and hardware platform were kept unchanged. Table 5 reports the mean and standard deviation of the main evaluation metrics. The improved YOLOv11s consistently outperforms the baseline across the three runs. On the URPC dataset, the average mAP@0.5 reaches 88.4%, which is 3.2 percentage points higher than that of YOLOv11s. On the RUOD dataset, the average mAP@0.5 reaches 87.9%, exceeding the baseline by 1.9 percentage points. The small standard deviations indicate that the performance gains are stable and are not caused by random fluctuations in a single training run.

Table 6 provides a concise conceptual comparison between the proposed method and typical YOLO-based improvements. Conventional YOLO modifications mainly aim to improve general detection accuracy, inference speed, or feature fusion. In contrast, the proposed framework is designed according to the feature-level effects of underwater image degradation. SCConv is introduced to reduce spatial and channel redundancy; Shuffle Attention strengthens channel–spatial interaction under complex illumination and backgrounds; and *Focaler-IoU* improves localization for blurred, small, and partially occluded underwater targets. This degradation-aware design distinguishes the proposed framework from general module optimization and supports its practical use in underwater robotic detection.

#### 3.3.5. Ablation Experiment

To comprehensively evaluate the independent contributions and synergistic effects of the proposed submodules, a full-permutation ablation study was conducted. Table 7 details the performance across all eight possible configurations, where S stands for Shuffle Attention, D for Spatial Channel Reconstruction Convolution (SCConv), F for *Focaler-IoU*, and B represents the baseline YOLOv11s.

The experimental results demonstrate a clear progressive enhancement. Each independent module yields a positive gain over the baseline, and crucially, any dual-module combination consistently outperforms its single-module counterparts. This indicates strong compatibility and an absence of feature-extraction conflict among the mechanisms. Ultimately, the simultaneous integration of all three modules achieves an optimal *mAP* of 88.4%, confirming their robust synergistic effect in maximizing overall detection performance.

#### 3.3.6. Edge Device Deployment and Power Consumption Analysis

To evaluate the practical deployment feasibility of the improved YOLOv11s model on resource-constrained underwater platforms, inference tests were conducted on a representative edge computing device, the NVIDIA Jetson Xavier NX (16 GB). Given the stringent energy and computational constraints of ROVs, the PyTorch model was converted into a TensorRT engine to enable hardware-specific optimization. During the evaluation, the jtop utility was employed to monitor real-time hardware utilization and power consumption. The software environment included Python 3.8.10, PyTorch 2.0.0, CUDA 11.8, TensorRT 8.6.1, Ultralytics YOLO 8.3.163, and jtop 4.2.4.

As summarized in Table 8, the improved model achieves an inference speed of 32.5 FPS with an average power consumption of 12.4 W. Although integrating SCConv, Shuffle Attention, and *Focaler-IoU* introduced a marginal computational overhead compared to the baseline YOLOv11s (36.2 FPS, 11.5 W), the inference speed remains comfortably above the 30 FPS real-time processing threshold. This minor efficiency trade-off is fully justified by the substantial improvements in detection accuracy in complex underwater environments. Ultimately, these metrics confirm that the proposed framework satisfies the stringent power and computational constraints of underwater robotic missions, proving its viability for direct onboard deployment.

#### 3.3.7. Underwater Robot Prototype Grasping Experiment and Results Analysis

(1) Artificial Pond Experiment

**Figure 20:**
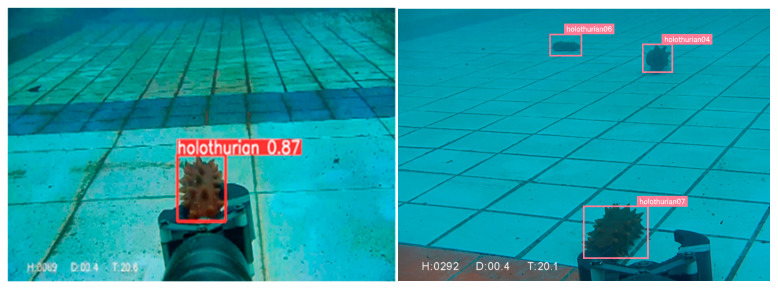
ROV tank target detection results.

Figure 20 presents the results of the detection experiments conducted in the author’s artificial pool test. The experiments revealed that, in addition to the wide variety of targets, the ROV frequently encountered occlusion and overlapping phenomena during multi-target recognition. Nevertheless, it could still detect multiple targets simultaneously.

(2) Natural Lake Water Experiment

**Figure 21:**
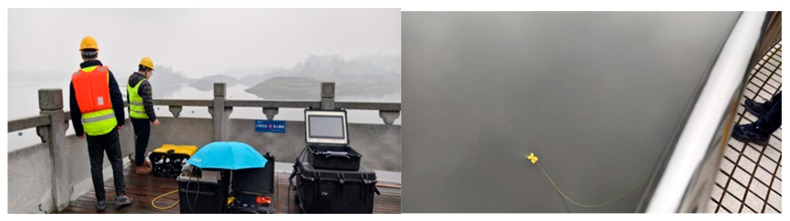
Natural lake water experiment.

This study selected Dengbu Island in Zhoushan City, Zhejiang Province, for conducting real-water detection experiments. The seas offer a variety of detection conditions and depths for underwater target-detection tests. The region’s variety of sediment types makes it easier to test and validate the dependability and performance of underwater detection equipment across various sedimentary settings. Figure 21 presents the natural lake water experiment.

**Figure 22:**
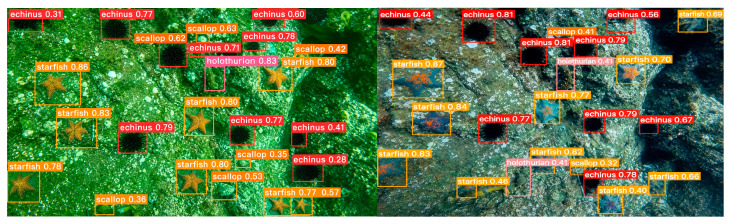
Comparison for complex background detection.

**Figure 23:**
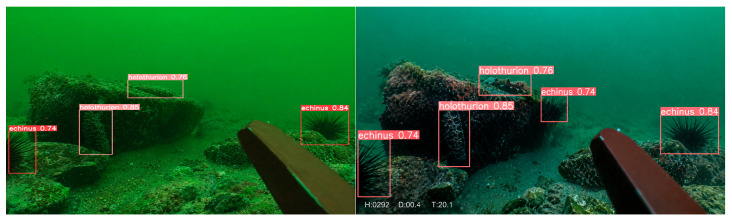
Comparison for occlusion environment detection.

**Figure 24:**
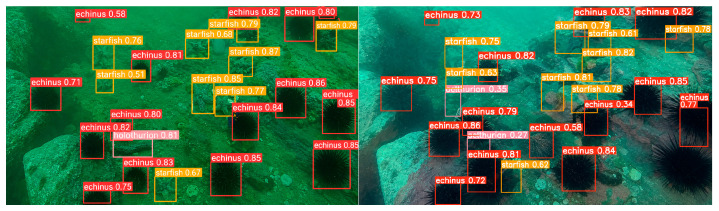
Comparison for underwater multi-class dense target detection.

To evaluate the detection performance of this algorithm, representative scenarios covering diverse conditions were selected, including complex underwater environments, occluded scenes, and target-dense regions. As shown in Figure 22, Figure 23 and Figure 24, the network was compared with the improved YOLOv11s. The left images depict performance before enhancement, while the right images show performance after enhancement.

The background in Figure 22 is mainly composed of rocks with colors similar to those of sea cucumbers, while the white patches on the rocks closely resemble scallops. This makes it difficult to distinguish sea cucumbers and scallops from the surrounding background. The original YOLOv11s model misidentifies some sea cucumbers as other objects, indicating its limited robustness in complex underwater backgrounds. In contrast, the improved YOLOv11s model provides more accurate detection results in this scenario. Figure 23 shows an underwater scene with blurred targets and partial occlusion, where the original model fails to detect the target reliably. The improved model correctly identifies these targets. This improvement can be attributed to the enhanced feature reconstruction and channel–spatial interaction introduced by SCConv and Shuffle Attention, as well as the improved localization ability provided by *Focaler-IoU*. As shown in Figure 24, the improved model also achieves more accurate recognition of overlapping and small targets, whereas the baseline YOLOv11s shows missed detections in densely packed scenes.

However, it must be noted that the superiority and robustness of the proposed framework demonstrated above are strictly bounded by the experimental conditions analyzed in this study. The performance validations are specifically limited to the shallow-water optical degradations represented by the URPC and RUOD datasets (e.g., typical turbidity and specific color distortion), the selected benthic target categories, and the computational constraints of the edge device used. Therefore, the performance advantages highlighted in this section should be interpreted exclusively within the context of these specific scenarios, rather than being broadly generalized to extreme deep-sea environments with zero visibility or severe artificial lighting interference.

## 4. Conclusions

This study presents an enhanced YOLOv11s-based detection framework specifically optimized for underwater robotic missions. By integrating SCConv, Shuffle Attention, and *Focaler-IoU*, the proposed model effectively addresses typical underwater degradations while maintaining high inference efficiency on edge platforms. Extensive experiments on the URPC and RUOD datasets demonstrate that the proposed model achieves a favorable balance between detection accuracy and inference efficiency, reaching 88.4% mAP@0.5 and 87 FPS on the URPC dataset.

Despite these results, the current research is primarily focused on specific shallow-water optical environments and benthic target categories. The model’s performance in extreme deep-sea scenarios with near-zero visibility or severe artificial lighting interference remains an area for further definition. Additionally, the inherent physical limitations of optical sensors in highly turbid waters present a significant challenge for single-mode perception frameworks.

Future research will focus on two primary trajectories. First, we intend to broaden the training corpus to encompass a more diverse range of underwater environments, thereby enhancing the model’s ecological generalization. Second, we will investigate multi-modal sensor fusion techniques—specifically the integration of acoustic sonar data with optical imagery—to achieve more reliable all-weather target recognition for underwater robots in complex marine engineering applications.

## Figures and Tables

**Table 1:** Dataset partitions used in the experiments.

Dataset	Total Images	Categories	Training	Validation	Split Ratio
URPC	5543	4	4989	554	9:1
RUOD	13,950	10	12,555	1395	9:1

**Table 2:** Simple parameter configurations.

Parameter	Value
Optimizer	SGD
Batch size	4
Learning rate	0.01
Confidence threshold	0.5
Weight decay coefficient	0.0005
Maximum epochs	100
Early stopping criterion	Best validation performance

**Table 3:** Comparison of different models on the URPC dataset.

Model	*AP* (%)				mAP@0.5 (%)	FPS
	Sea Urchin	Sea Cucumber	Star Fish	Scallop		
SSD	74.7	69.9	75.2	60.2	70.0	21
YOLOv8s	88.1	73.6	87.1	86.0	83.7	92
YOLOv9s	88.9	74.1	87.6	86.2	84.2	88
YOLOv10s	89.2	74.5	87.9	86.6	84.6	91
RT-DETR-R18	85.5	73.8	87.4	86.1	84.0	74
YOLOv12s	90.5	75.3	88.5	87.1	85.4	85
DPT-YOLO	87.2	74.2	86.2	85.3	84.1	72
LGM-YOLOv11	90.2	74.8	88.7	86.2	85.9	82
YOLOv11s	89.8	75.1	88.3	84.9	85.2	90
Improved YOLOv11s	92.1	76.6	89.1	87.4	88.4	87

**Table 4:** Comparison of different models on the RUOD dataset.

Model	*Precision* (%)	*Recall* (%)	mAP@0.5 (%)	FPS
SSD	72.6	68.9	70.8	22
YOLOv8s	85.0	82.1	84.8	92
YOLOv9s	85.7	82.6	85.2	88
YOLOv10s	86.0	82.9	85.6	91
RT-DETR-R18	85.1	81.7	84.7	73
YOLOv12s	87.0	83.8	86.6	85
DPT-YOLO	84.8	81.6	84.3	71
LGM-YOLOv11	86.5	83.4	86.2	81
YOLOv11s	86.2	83.1	86.0	90
Improved YOLOv11s	88.1	85.0	87.9	87

**Table 5:** Statistical validation over three independent runs.

Dataset	Model	*Precision* (%)	*Recall* (%)	mAP@0.5 (%)
URPC	YOLOv11s	86.1 ± 0.3	82.5 ± 0.3	85.2 ± 0.2
URPC	Improved YOLOv11s	89.0 ± 0.3	85.4 ± 0.3	88.4 ± 0.2
RUOD	YOLOv11s	86.2 ± 0.3	83.1 ± 0.3	86.0 ± 0.2
RUOD	Improved YOLOv11s	88.1 ± 0.2	85.0 ± 0.3	87.9 ± 0.2

**Table 6:** Conceptual comparison with typical YOLO-based improvements.

Aspect	Typical YOLO Improvements	Proposed Method
Motivation	Accuracy/speed/fusion	Underwater degradation
Design logic	Generic module optimization	Degradation-aware adaptation
Key focus	Feature enhancement	Redundancy, attention, localization
Validation	General comparison	URPC, RUOD, robotic tests

**Table 7:** Ablation experiment results on the underwater datasets.

Model	B	S	D	F	mAP@0.5 (%)	FLOPs (G)	FPS	Parameter (M)
1	√				85.2	27.7	95.9	10.9
2	√	√			86.1	31.0	90.2	13.4
3	√		√		86.7	31.6	89.1	13.9
4	√			√	86.3	31.8	89.2	14.1
5	√	√	√		87.1	32.6	88.7	16.4
6	√	√		√	86.9	32.4	88.3	16.1
7	√		√	√	87.2	32.6	87.1	16.6
8	√	√	√	√	88.4	32.9	86.7	17.6

Note: √ indicates that the corresponding component is included in the model. B, S, D, and F denote the baseline YOLOv11s, Shuffle Attention, SCConv, and *Focaler-IoU*, respectively.

**Table 8:** Comparison of inference speed and power consumption on the edge device.

Model	Inference Speed (FPS)	Average Power (W)
YOLOv11s	36.2	11.5
Improved YOLOv11s	32.5	12.4

## Data Availability

The dataset used in the paper can be downloaded here: https://openi.pcl.ac.cn/OpenOrcinus_orca/URPC2020_dataset/datasets (accessed on 10 March 2022).
